# A novel cell-based transplantation method using a Rho kinase inhibitor and a specific catheter device for the treatment of salivary gland damage after head and neck radiotherapy

**DOI:** 10.1016/j.bbrep.2022.101385

**Published:** 2022-11-12

**Authors:** Atsushi Kasamatsu, Reo Fukushima, Koki Nakamura, Kohei Kawasaki, Shusaku Yoshimura, Tomoyoshi Koyama, Chonji Fukumoto, Isao Miyamoto, Katsuhiro Uzawa

**Affiliations:** a Department of Dentistry and Oral-Maxillofacial Surgery, Chiba University Hospital, 1-8-1 Inohana, Chuo-ku, Chiba, 260-8670, Japan; b Department of Oral Science, Graduate School of Medicine, Chiba University, 1-8-1 Inohana, Chuo-ku, Chiba, 260-8670, Japan; c Department of Oral and Maxillofacial Surgery, Dokkyo Medical University, School of Medicine, 880 Kitakobayashi, Mibu, Shimo-Tsuga, Tochigi, 321-0293, Japan

**Keywords:** Radiotherapy, Head and neck cancer, Salivary gland, Cell-based transplantation, Rho kinase inhibitor, Auto-transplantation

## Abstract

Radiotherapy (RT) for head and neck cancer results in irreversible damage to salivary glands (SGs) and decreases saliva production, leading to a dry mouth. To date, there are no satisfactory therapies to solve this problem. We recently established a novel culturing method using a Rho kinase inhibitor (RI), Y-27632, that maintained cellular morphology and function for a prolonged period of time. In the present study, we investigated whether cell-based transplantation using our culturing method ameliorated the dysfunction of irradiated SGs. First, rat SG cells were cultured in a medium with RI. Cells were characterized by morphological findings and mRNA expression analysis. We also assessed features of SG cells in three-dimensional (3-D) culture by scanning electron microscopy and immunohistochemistry (IHC). The RI-containing medium led to higher cell proliferation of rat SG cells with preservation of cell morphology and higher alpha-amylase (AMY) expression in both 2-D and 3-D culture systems. To establish the atrophic-SG models, external RT at a dose of 15 Gy was delivered to the head and neck fields of nude rats. The SG cells derived from GFP-rats were cultured in medium with RI, after which they were transplanted into the submandibular glands of atrophic-SG rats using a catheter placed into Wharton's duct. IHC and salivary flow rate (SFR) analyses were measured 12 weeks after the transplantation. Following transplantation, donor cells (GFP-SG cells) were primarily located in the ductal region of the SG, and AMY expression in SGs and the SFR were increased in the SG cell transplantation group compared with the control. Those data indicated that cell-based therapy using RI-treated SG cells could restore salivary hypofunction of irradiated SGs by direct integration of the donor cells in the duct of SGs. We propose that these data support future clinical plans in which SG cells would be excised from the labial minor SGs of the patients with head and neck cancers prior to RT, cultured during RT, and auto-transplanted into SGs using a catheter into the Wharton's duct. We believe that our culturing and transplantation methods can be applied to SG cells, constituting a therapeutic approach for the treatment of patients with dry mouth after not only RT but also aging and Sjögren's syndrome.

## Highlights

• We cultured SG cells for a prolonged period of time using a Rho kinase inhibitor.
• We established a novel SG transplantation method through Wharton's duct.
• SG cell transplantation ameliorated salivary dysfunction of atrophic-SG rats.
• Salivary flow rates were significantly increased after SG cell transplantation.
• Our method constitutes a therapeutic approach for head and neck cancer patients.

## 1 Introduction

Dry mouth due to hypofunction of salivary glands (SGs) is the most significant long-term complication of radiotherapy (RT) for head and neck cancers [1]. Dry mouth shows various symptoms, including active dental caries, oral candidiasis, and difficulty with eating, swallowing, or speaking [2]. Patients with dry mouth have been treated with salivary substitutes or medications, such as pilocarpine and cevimeline hydrochloride [3]. These treatments temporarily relieve the patients’ symptoms; however, no treatment is available for regenerating atrophic-SGs.

We previously revealed that a chemical compound, Y-27632, which is a highly selective Rho kinase inhibitor (RI), increases colony-forming efficiency of several types of cells [4]. Other studies had reported that RI inhibited cellular apoptosis through the cadherin-dependent pathway [[5], [6], [7], [8], [9]]. Those data suggested that cellular differentiation is inhibited and that cellular proliferative capacity and senescence may improve after RI treatment. Standard RT for head and neck cancer patients is hyperfractionated, which needs 6–7 weeks for completion of treatment [10]. For autologous SG transplantation to the patients, we need to not only avoid RT damage but also culture the SG cells for an extended time during RT. Therefore, we used an RI to accelerate proliferation and enhance the lifespan of SG cells in culture.

Gene therapy and stem cell transplantation for SG regeneration have been reported by several groups [11,12]. Gene therapy for the treatment of SG dysfunction shows promise as a future treatment, as gene transfer to irradiated SGs has been shown to increase saliva production in animal models [13]. However, clinical application of gene therapy to treat atrophic-SGs requires improved gene delivery systems. Moreover, with currently available systems, there are concerns regarding safety, efficiency and the duration of transgene expression. On the other hand, preclinical and clinical trials using stem cells for SG regeneration were recently reported [[14], [15], [16], [17]]. Stem cells derived from bone marrow or adipose tissue are potential sources of stem cells for the treatment of salivary hypofunction [[18], [19], [20], [21]]. In stem cell-based therapies, stem cells have been transplanted via intravenous injection or direct injection to SGs using a fine needle with ultrasound guidance. However, these methods have been accompanied by some complications for recipients, such as side effects in other organs and infection. In the present study, we established a novel transplantation method using a catheter to avoid those problems.

We undertook the present study to determine whether focal administration of SG cells through the Wharton's duct of the SG could ameliorate RT-induced SG damage. Here, we demonstrate the efficacy of this novel technique, using RI treatment and catheter-mediated cell transplantation into irradiated atrophic-SG. In conclusion, we believe that our methods should be therapeutic sources for SG transplantation for human patients with head and neck cancer after RT but also treatments for aging and Sjögren's syndrome.

## 2 Materials and methods

### 2.1 Ethics statement

The Ethical Committee of Chiba University approved the current study protocol (approval number, 27–101 and 680). The study was performed in accordance with the ethical standards of the Declaration of Helsinki. All rats were kept under clean conventional conditions at the Chiba University Animal Resource Center. Our animal experiments were performed in accordance with the Guide for Animal Experimentation of Chiba University. All healthy volunteers provided written informed consent before inclusion in the study.

### 2.2 Cell culture

SG tissues were obtained from the submandibular glands of Sprague Dawley rats and GFP-rats (W-Tg(CAG-EGFP)3 Ys) (CIEA, Tokyo, Japan). Human labial minor SGs from three healthy volunteers were excised at the Dentistry and Oral-Maxillofacial Surgery, Chiba University Hospital. The cells were washed three times with cold phosphate buffered saline (PBS) containing 0.5% gentamicin (Sigma-Aldrich, St. Louis, MO, USA) until the surface was free of blood or other material. The connective tissues were carefully removed using fine scissors. The remaining tissues were minced using a clean No. 11 scalpel blade to produce fragments smaller than 0.5 mm^3^. The tissues were then treated with collagenase B (2.5 mg/mL), elastase (1 mg/mL), and DNase I (1 mg/mL) (all from Roche Diagnostics GmbH, Mannheim, Germany) at 37 °C for 30 min with gentle shaking. The digested tissue suspension was filtered through 100-μm cell strainers. Collection tubes were centrifuged at 300 g for 5 min. The supernatant was discarded and the cell pellets were resuspended in the culture medium according to our previous report [4]. The medium consisted of Dulbecco's modified Eagle's Medium (DMEM)-low glucose (GIBCO-BRL, Life Technologies, Gaithersburg, MD, USA), 10% fetal bovine serum (FBS), and 0.5% gentamicin, with/without 10 μM RI, Y-27632 (Fujifilm Wako, Saitama, Japan). The cells were seeded homogenously into 60-mm cell culture dishes (Corning, Steuben, NY, USA) and cultured at 37 °C in a 5% CO_2_ atmosphere in a humidified incubator. The medium was subsequently changed every 3 days.

We used Matrigel basement membrane matrix (BD Biosciences, Franklin Lakes, NJ, USA) to investigate the effect of a three-dimensional (3-D) culture system on rat SG cells. When the cells reached more than 70% confluence, they were harvested for 3-D cultures. Aliquots of cells (5 × 10^4^ cells) in the culture medium (300 μL) were added to 300 μL of cold Matrigel and the mixture was seeded into 4-well glass chamber slides (BD Biosciences). The slides were incubated at 37 °C for 30 min to allow gel formation. The medium was changed every 3 days.

### 2.3 Quantitative reverse transcriptase-polymerase chain reaction (qRT-PCR)

Primer 3Plus (online free software, http://primer3plus.com/) was used to design the gene-specific primers. The sequences of the designed primers were as follows: rat amylase (rat *Amy*, forward, 5′- TTGTCGTCTGTCGGCCTTC -3′; reverse, 5′-CACTGCTTCACCACCCAGAT-3′), human *AMY* (forward, 5′-GATAATGGGAGCAACCAAGTGGC-3′; reverse, 5′-CAGTATGTGCCAGCAG11GAAGAC-3′), rat glyceraldehyde-3-phospate dehydrogenase (rat *Gapdh*, forward, 5′-CAACTCCCTCAAGATTGTCAGCAA-3′; reverse, 5′-GGCATGGACTGTGGTCATGA-3′), and human *GAPDH* (forward, 5′-AGCCACATCGCTCAGACAC-3′; reverse, 5′-GCCCAATACGACCAAATCC-3′). The normalization of the transcript levels of target genes was previously described [[22], [23], [24]].

### 2.4 Scanning electron microscopy (SEM)

After 12 days of culture in Matrigel, the samples were fixed with 2.5% glutaraldehyde and washed with PBS before post-fixation in 1% OsO_4_ and washing with deionized-distilled H_2_O. The samples were dehydrated with solutions having increasing alcohol concentrations, treated with propylene oxide, and embedded in epoxy resin. The slides were dried using liquefied CO_2_. The specimens were coated with conductive platinum with a precision etching coating system model 1682 (Gatan Inc., Pleasanton, CA, USA) and examined by SEM using a JSM-7001 F microscope (JEOL, Ltd., Tokyo, Japan).

### 2.5 Histological examination

Paraffin sections (4 μm) were prepared for staining with HE and PAS and immunohistochemistry (IHC). Briefly, after deparaffinization and hydration, the endogenous peroxidase activity was quenched by a 3-min incubation in 0.3% hydrogen peroxide in 100% methanol. For HE and PAS staining, the sections were stained as described previously [25]. For IHC, the sections were incubated with goat anti-AMY polyclonal antibody (Santa Cruz Biotechnology, Dallas, TX, USA), rabbit anti-mucin1 polyclonal antibody (LifeSpan BioScience, Seattle, WA, USA), rabbit anti-aquaporin 5 polyclonal antibody (AQP5; Abcam, Cambridge, UK), or rabbit anti-Na^+^-K^+^-2Cl^-^ cotransporter isoform 1 polyclonal antibody (NKCC1, a marker for salivary acinar cells, Abcam) at 4 °C in a moist chamber overnight. After treatment with the primary antibodies, the specimens were washed with PBS and treated with anti-goat (Invitrogen, Carlsbad, CA, USA) or anti-rabbit (Abcam) IgG horseradish peroxidase as a secondary antibody for 2 h at room temperature and then washed three times with tris-buffered saline. The slides were subsequently treated with a peroxidase substrate kit DAB (Vector Laboratories, Burlingame, CA, USA) and lightly counterstained with hematoxylin, dehydrated with ethanol, cleaned with xylene, and mounted.

Submandibular glands of rats were dissected in cold PBS, fixed in 4% paraformaldehyde overnight, and mounted in OCT compound (Miles, Elkhart, IN) on dry ice as described previously [26]. The samples were then stored at −80 °C until the time for cryosectioning. Tissue blocks were sectioned using a cryostat (CM1860 UV; Leica, Wetzlar, Germany). The frozen sections from the submandibular glands of the rats were incubated overnight with an Alexa Fluor 488-conjugated rabbit polyclonal anti-GFP antibody at 4 °C (Life Technology, Carlsbad, CA, USA) to detect GFP. To visualize cell nuclei, the cells were counterstained with DAPI (Vector Laboratories). Images were captured from each surface using a Keyence BZ-X800 microscope with Keyence software (Osaka, Japan).

For immunocytochemistry (ICC), the cells were fixed in 0.1% paraformaldehyde, 2 mM MgCl_2_, and 1 mM EDTA in culture medium at room temperature for 20 min, washed twice with culture medium, and permeabilized with culture medium containing 0.5% Triton X-100 at room temperature for 10 min. The cells were washed twice with culture medium and blocked with culture medium containing 5% BSA (Gibco-BRL, Waltham, MA, USA) for 30 min. The cells were incubated with anti-AMY antibody (Santa Cruz Biotechnology) for 1 h, washed three times with culture medium, and then incubated with anti-goat secondary antibody (Alexa Fluor® 647, Abcam) for 1 h. To visualize cell nuclei, the cells were counterstained with DAPI. Images were captured from each surface using a Keyence BZ-X800 microscope with Keyence software.

### 2.6 Rat atrophic-SG models

Nude rats (F344-NJcl-rnu/rnu, 8 weeks of age) were subjected to SG damage by local head and neck irradiation exposure to 15 Gy (Philips CMG 41 X, 200 kV, 10 mA, 5 Gy/min) according to a previous report [16]. Briefly, the rats were anesthetized with medetomidine hydrochloride at a dose of 0.3 mg/kg (Meiji Seika Pharma, Tokyo, Japan), midazolam at a dose of 4.0 mg/kg (Astellas, Tokyo, Japan), and butorphanol at a dose of 5.0 mg/kg (Meiji Seika Pharma) solution given intraperitoneally [27]. The rest of the body was shielded with 3 mm of lead to reduce the beam strength (Fig. 3A).

### 2.7 Transplantation of SG cells into rat atrophic-SG models

In the rat atrophic-SG model, we used 8-week-old females as recipients of transplanted cells. Briefly, prior to the transplantation, the RI-treated SG cells from female GFP-rats were detached from culture dishes by enzymatic treatment with 0.05% trypsin/EDTA. The cell pellets were resuspended in the culture medium. The cell suspension (100 μL) containing 1 × 10^6^ cultured cells with 100 μL atelocollagen, 3-D scaffolds (Koken, Tokyo, Japan) was injected into the submandibular gland of the recipient rats through the Wharton's duct using a 3-Fr Atom-tube (Atom Medical, Tokyo, Japan) (Fig. 3B). A total of 15 animals were randomly divided into three groups: a non-irradiated group (Normal group), an irradiated and atelocollagen-injected group (RT group), and an irradiated and SG cell-transplantation group (RT + SG cell group). The submandibular glands were excised 12 weeks after the transplantation.

### 2.8 Salivary flow rate

Salivary secretory function was determined by measuring salivary flow rate (SFR) 12 weeks after the transplantation. Saliva was collected from the floor of the mouth using a micropipette 5 min after stimulation by intraperitoneal injection of pilocarpine (2 mg/kg). Collected saliva was placed in pre-weighed 1.5 mL microcentrifuge tubes and SFR (mL/minutes) was calculated by dividing the weight (mg) of all saliva collected by the collection time (min).

### 2.9 Establishment of transplantation method for human submandibular gland using a catheter

Under local anesthesia with xylocaine, a lacrimal probe was introduced to dilate a sublingual caruncle, the entrance of Wharton's duct, of the submandibular gland. After the orifice was sufficiently dilated, an adult intravenous catheter (3-Fr Atom-tube) was introduced (Fig. 4D). Then, 1 mL of contrast material, an iodinated water soluble agent, was injected to see whether catheter insertion was correctly placed. After the injection with the contrast material, a radiograph of the SG was taken.

### 2.10 Statistical analysis

Each experiment was repeated at least three times. The data were analyzed using an unpaired *t*-test or one-way analysis of variance (ANOVA), followed by a post hoc Tukey's test (ANOVA with Tukey's multiple comparison test). All statistical analyses were performed with Microsoft Excel (Microsoft, Redmond, WA, USA). The data are expressed as the mean ± standard error of the mean.

## 3 Results

### 3.1 Culture of rat SG cells with/without RI

**Fig. 1:**
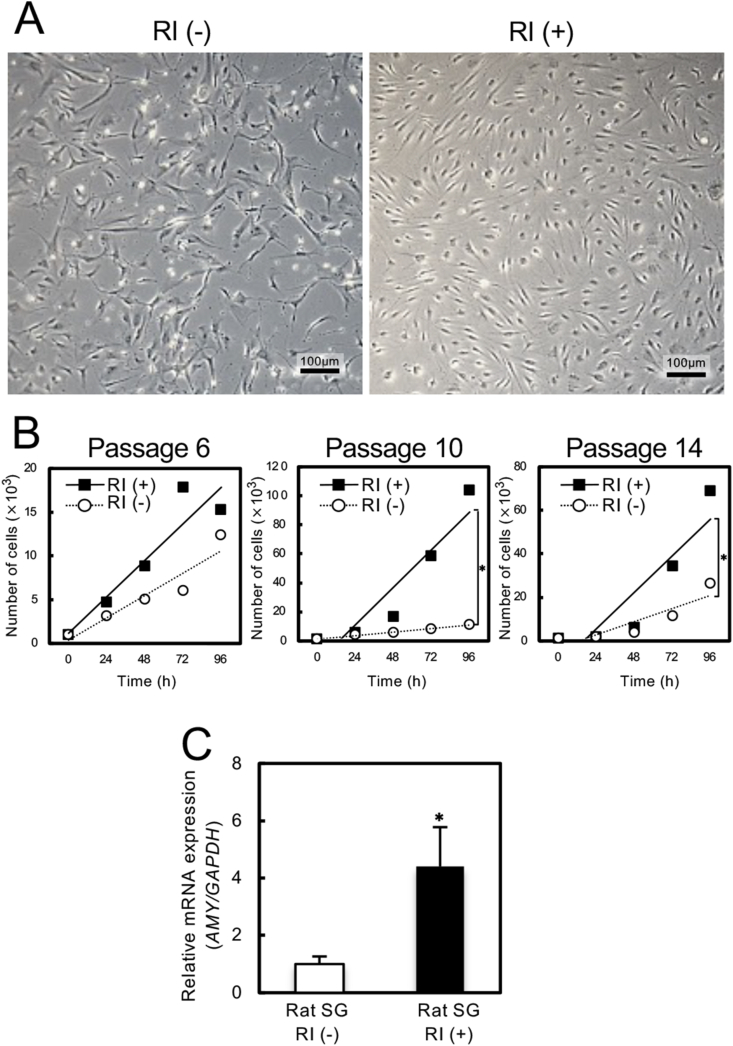
Culture of rat SG cells with/without RI. **A**, Cell morphologies changed with continued growth in the absence of RI, but not in the presence of RI (passage-10). **B**, Cellular growth of rat SG cells with/without RI. The cellular proliferation assay demonstrated that RI-treated SG cells retained higher cell growth rates than did the non-RI culture condition (*, *P* < 0.05). **C**, *Amy* mRNA expression in rat SG cells with/without RI. *Amy* mRNA expression was significantly upregulated in the RI-treated SG cells compared with the cells cultured without RI (passage-10; *, *P* < 0.05; N = 3).

To investigate the effects of RI on cultivation of rat SG cells, we examined cell morphology and followed cell proliferation using RI-treated SG cells. Cell morphologies changed as the number of passage increased under non-RI culture condition but not in the RI-treated SG cells (Fig. 1A). The cellular proliferation assay demonstrated that the RI-treated SG cells possessed a higher cell growth rate than did those in the absence of RI (Fig. 1B). To analyze the mRNA expression status of *Amy*, rat SG cells cultured with/without RI were subjected to qRT-PCR. *Amy* mRNA expression was significantly upregulated in the RI-treated SG cells compared with the cells cultured without RI after 10 passages (Fig. 1C, *P* < 0.05).

### 3.2 Characterization of rat SG cells in a 3-D culture system

**Fig. 2:**
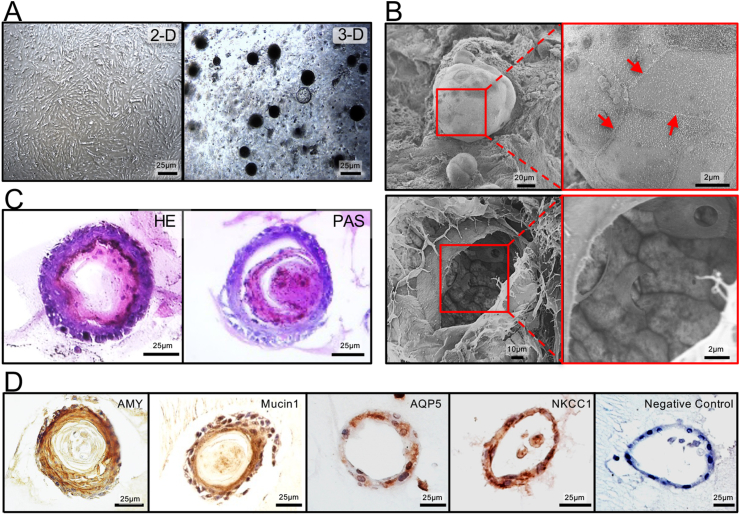
Characterization of rat SG cells in a 3-D culture system. **A,** Rat SG cells were tightly connected and exhibited a typical cobblestone morphology in a 2-D culture system (2-D). Rat SG cells were cultured in Matrigel to permit formation of 3-D structures. These cells formed rounded clumps in certain areas of the Matrigel (3-D). **B,** SEM analysis of the 3-D structure of cultivated rat SG cells. The cell clumps were embedded in Matrigel (upper panel), and high magnification showed that the clumps were composed of closely associated cells (arrows in the upper panel). The hollow lumen structure and tightly connected cells aggregates were observed through a crack in the cell clump (lower panel). **C,** HE and PAS staining of rat SG cells. HE staining showed that the cell clumps that comprised two to three layers of SG cells, included luminal spaces and several cells were localized in those spaces (HE). The internal structure of the cell clumps stained positively for PAS, suggesting the presence of glycoproteins, carbohydrates, and mucins (PAS). **D,** IHC of rat SG cells. The cell clumps stained positively for AMY and mucin1, markers of mucopolysaccharides. Positive staining was also observed for AQP5 and NKCC1, but the localizations differed from normal SG tissues.

**Fig. 3:**
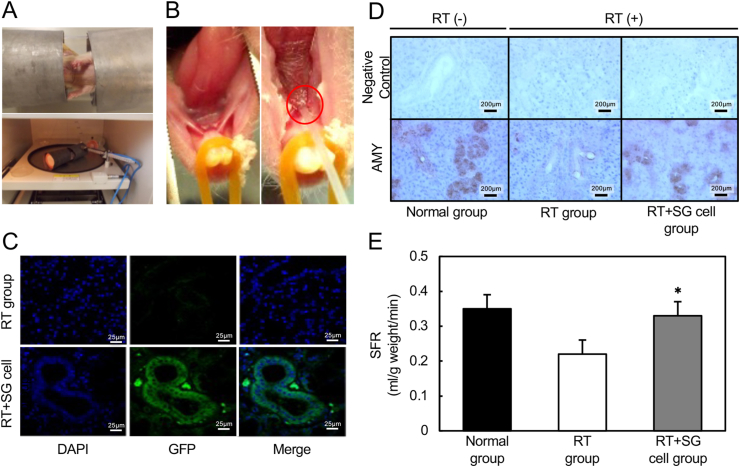
Transplantation of GFP-rat SG cells to irradiated SG through the Wharton's duct. **A**, Establishment of irradiated SG rat models. Anesthetized rats were immobilized in a tube shielded with lead. Only the head and neck regions were exposed. The nude rats were locally irradiated in the head and neck regions with a single dose of 15 Gy. **B**, The catheter was inserted into Wharton's duct from the sublingual caruncle (red circle) to the submandibular gland. **C**, IHC of transplanted SGs was performed using anti-GFP antibody. GFP-positive cells were observed in the duct of the RT + SG cell group. **D**, AMY expression after RT in SGs. Low AMY expression was found in the RT group. AMY expression was recovered in the RT + SG cell group 12 weeks after the transplantation. **E**, SFR after the transplantation. SFR was significantly increased in the RT + SG cell group 12 weeks after the transplantation with SG cells (N = 5). (For interpretation of the references to colour in this figure legend, the reader is referred to the Web version of this article.)

Cells were cultured in 60-mm culture dishes for 12 days. The cells were closely associated and exhibited a typical cobblestone morphology (Fig. 2A and 2-D culture). The cells were also cultured for 12 days in Matrigel to permit the formation of 3-D structures. These cells formed round clumps in certain areas of the Matrigel (Fig. 2, Fig. 3D culture). We performed SEM analysis to further investigate the morphologic characteristics of the cell clumps. Under high magnification, it was apparent that cell clumps in Matrigel were composed of tightly connected cells (Fig. 2B, upper panel, arrows). The hollow lumen structure and tightly connected cellular aggregation were observed through a crack in the cell clump (Fig. 2B, lower panel). We next stained the cell clumps with HE and PAS. The functionalities of acinar cells were studied by measurement of mucopolysaccharide production revealed by PAS staining. AMY production was assessed by IHC. HE staining showed that the cell clumps, which comprised two to three layers of SG cells, had luminal spaces and several cells localized within those spaces (Fig. 2C, HE). The internal structure of the cell clumps stained positively for PAS, suggesting the presence of glycoproteins, carbohydrates, and mucins (Fig. 2C, PAS). To further evaluate the characteristics of the cell clumps, we performed IHC with antibodies against AMY, mucin1, AQP5, and NKCC1. The cell clumps stained positively for AMY and mucin1, markers of mucopolysaccharides (Fig. 2D). Positive staining was also observed for AQP5 and NKCC1 (Fig. 2D), but the localization differed from that seen in normal SG tissues [28,29].

**Fig. 4:**
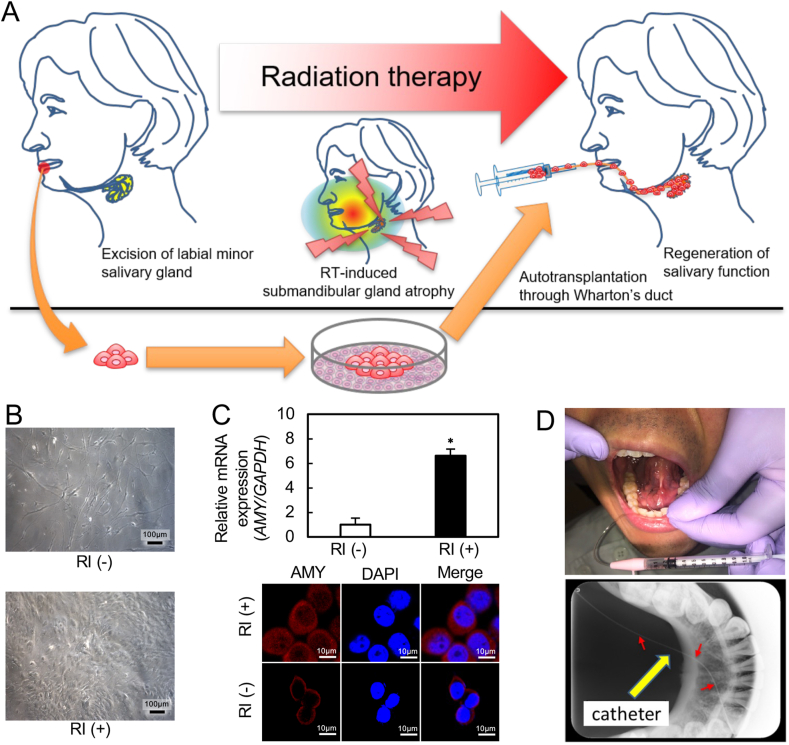
Plans for SG auto-transplantation of human patients. **A**, Schematic diagram of human application shows that some labial minor SGs are extracted prior to RT and cultured during RT. Auto-transplantation of cultured SG cells through Wharton's duct into the atrophic submandibular gland after RT is performed. **B**, Morphology of human SG cells with/without RI. The human SG cells cultured with RI showed similar cell growth and cell morphology to those of rat SG cells. **C**, AMY expression in human SG cells with/without RI. *AMY* mRNA expression was significantly upregulated in RI-treated SG cells compared with non-RI culture condition (passage-10; *, *P* < 0.05; N = 3). The ICC data also showed strong immunoreactivity of AMY in the RI-treated SG cells, whereas SG cells cultured without RI showed weak immunoreactivity of AMY. **D**, Transplantation method was confirmed using a catheter device through Wharton's duct in healthy volunteers. Proper insertion of the catheter was confirmed by radiograph.

### 3.3 Transplantation of GFP-rat SG cells to irradiated SGs via Wharton's duct

We first established a rat atrophic-SG model. Briefly, anesthetized rats were immobilized in a tube shielded with lead such that only the head and neck regions were exposed. The exposed regions of the nude rats were irradiated with a single dose of 15 Gy (Fig. 3A). Then, to insert the catheter, we placed a 3-Fr Atom-tube into the Wharton's duct from the sublingual caruncle for the submandibular gland (Fig. 3B). The rats' upper incisors were locked on a wire, and the lower incisors were hooked using a rubber string in order to hold the mouth open (Supplementary Fig. 1). To verify the insertion of the catheter into the submandibular gland through Wharton's duct, a trypan blue solution was injected through the catheter. We observed a stained submandibular gland, indicating that the transplantation methods were satisfactory (Supplementary Fig. 2). Then, to assess the localization of the transferred donor cells (GFP-SG cells) in the rat atrophic-SG, we performed IHC of recipient SGs using GFP antibody. GFP-positive cells were observed in the duct of SGs in the RT + SG cell group (Fig. 3C), indicating that the transplanted SG cells were incorporated into the duct of the SG. To investigate the SG function after the transplantation, we examined the AMY expression level. Although low AMY expression was found in the RT group, higher AMY expression was recovered in the RT + SG cell group (Fig. 3D). We then measured the SFR in the Normal, RT, and RT + SG cell groups. SFR was significantly increased in the RT + SG cell group after the transplantation compared with the RT group (Fig. 3E).

### 3.4 SG transplantation for patients with SG damage after head and neck RT

Fig. 4A illustrates our plans for conducting auto-transplantation of patients with SG damage after head and neck RT. The approach is based upon our cultivation method and the use of the catheter described above. For human application, labial minor SGs would be extracted prior to RT and cultured with RI to increase cell numbers. After cultivation, cells would be auto-transplanted through Wharton's duct into the atrophic submandibular gland. In pursuit of this plan, we first investigated the effect of RI on human SG cells from labial minor SGs. The human SG cells that were cultured with RI showed growth and cell morphology similar to that observed with rat SG cells (Fig. 4B). To analyze the expression status of AMY, we performed qRT-PCR and ICC in the RI-treated human SG cells. *AMY* mRNA expression was significantly upregulated in the RI-treated SG cells compared with non-RI culture conditions at passage-10 (Fig. 4C; *P* < 0.05). The ICC data also showed strong immunoreactivity of AMY in the RI-treated SG cells, whereas the SG cells cultured without RI showed weak immunoreactivity of AMY (Fig. 4C). Catheter insertion into Wharton's duct was performed using adult healthy volunteers. Proper insertion of the catheter was confirmed by radiographs, suggesting that transplantation of SG cells was entirely feasible (Fig. 4D). Therefore, our auto-transplantation method using labial minor SG cells should be effective for patients who have undergone RT for head and neck cancer.

## 4 Discussion

We previously showed that oral ingestion is closely related to plasma glucose concentration [30]. Saliva is also required for lubrication and oral homeostasis in addition to digestion [31]. RT for head and neck cancers results in irreversible damage to SGs and decreases saliva production. RT-induced hypofunction of the SGs usually occurs shortly after therapy [32]. A large number of head and neck cancer patients experience serious dry mouth after RT [33,34], suggesting that salivary hypofunction directly reduces the quality of life in such patients [31,35,36]. We suggest that regenerative therapy for SG is a logical approach to solve these problems.

Saito et al. reported that stem cells could be intravenously delivered to regions damaged by myocardial infarction to improve ventricular function in rat models [37]. The results indicated that circulating stem cells have the ability to improve the status of injured tissues. Lombaert et al. also reported that stem cells could ameliorate acinar cell loss and vascular damage of irradiated SGs [18,19]. Stem cells are considered to exert positive effects on functional restoration of several organs [[18], [19], [20], [21]]; however, the exact mechanisms by which stem cells improve their functions remain unclear. Reconstruction of an entire organ by tissue engineering is not feasible at present because in the case of the SG, it would require the regeneration of a complex ductal system, blood supply, and saliva production. In the present study, we investigated whether cell-based transplantation therapy using RI-treated SG cells, as donor cells, instead of stem cells, could restore the functions of irradiated SGs. Our results demonstrated that RI can accelerate the proliferation of SG cells, giving them the ability for long-term growth *in vitro* (Fig. 1). After transplantation of the rats, the donor SG cells were mainly observed in the ductal region of recipient irradiated SGs, and AMY expression and SFR were increased compared with the control group (Fig. 3).

There are currently two major delivery methods for transplantation of cells for SG regeneration: systemic intravenous injection and percutaneous direct injection into SG using ultrasound guidance [15,16,20,38]. Although both delivery methods lead to good recovery of SG function, their methods have safety concerns regarding non-targeted effects on other organs and delivery efficiency for the target organ. Here we presented a promising new approach using a catheter through Wharton's duct. Using this method, we can deliver the transplanted materials into SGs in rat and human models (Supplemental Fig. 2 and Fig. 4D).

Standard RT for patients with head and neck cancer is hyperfractionated, which needs 6–7 weeks for completion of treatment [10]. For autologous SG transplantation to the patients, we need to not only avoid RT damage but also culture the SG cells for an extended time during RT. To accommodate the requirements, we use RI for donor cell culture. Our future approach to human application includes collection of labial minor SGs prior to RT and cultivation with RI to increase cell numbers during RT. After the RT, we would auto-transplant the cultured SG cells through Wharton's duct into the atrophic submandibular gland (Fig. 4A). Although these methods are minimally invasive treatments, we need to monitor side effects after the auto-transplantation.

In conclusion, we propose here that cell-based therapy using RI-treated SG cells can restore the salivary function of irradiated SGs by direct integration of autologous donor cells into the duct of SGs. Therefore, we believe that our culturing and transplantation methods should be therapeutic sources for SG transplantation for human patients with head and neck cancer after RT but also treatments for aging and Sjögren's syndrome.

## Author contributions

A.K. and K.U. designed and directed the study. A.K., S.Y., TK, C.F., and I.M. performed the experiments. R.F., K.N., and K.K. contributed to the biochemical analyses, interpreted the data and the overall results. A.K. and K.U. reviewed the experimental data, drafted the manuscript and revised figures. All authors discussed the results and contributed to the final manuscript.

## Funding

This study was supported by 10.13039/501100001691JSPS KAKENHI grant numbers 18K09805, 19K19186, and 21K17128.

## Declaration of competing interest

The authors declare that they have no known competing financial interests or personal relationships that could have appeared to influence the work reported in this paper.

## Data Availability

The data that has been used is confidential.
