# Supplementary material for: A novel cell-based transplantation method using a Rho kinase inhibitor and a specific catheter device for the treatment of salivary gland damage after head and neck radiotherapy
Source: Biochem Biophys Rep. 2022 Nov 12;32:101385. doi: 10.1016/j.bbrep.2022.101385 (PMC9663336; doi:10.1016/j.bbrep.2022.101385)
Supplement: Multimedia component 1 [file mmc1.pdf]

Supplementary Figure 1

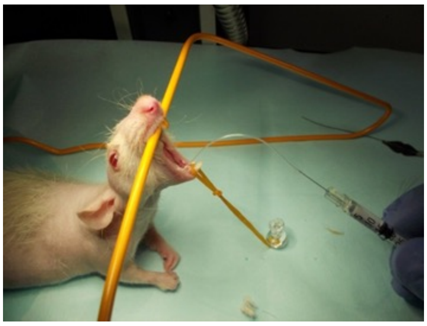

Supplementary Figure 1.
The rat upper incisors were locked on the wire, and the lower incisors were hooked using a rubber string in order to hold the mouth opening for insertion of catheter device.
