# Supplementary material for: A novel cell-based transplantation method using a Rho kinase inhibitor and a specific catheter device for the treatment of salivary gland damage after head and neck radiotherapy
Source: Biochem Biophys Rep. 2022 Nov 12;32:101385. doi: 10.1016/j.bbrep.2022.101385 (PMC9663336; doi:10.1016/j.bbrep.2022.101385)
Supplement: Multimedia component 2 [file mmc2.pdf]

Supplementary Figure 2

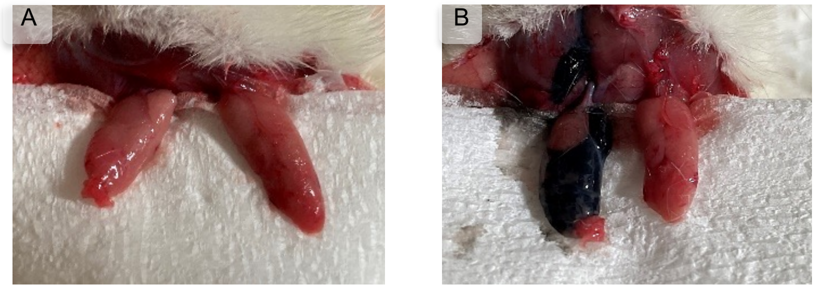

Supplementary Figure 2.
Trypan blue stain solution are injected through the catheter device to verify the procedure of the catheter insertion into the submandibular gland through Wharton’s duct.
